# RK-270D and E, Oxindole Derivatives from *Streptomyces* sp. with Anti-Angiogenic Activity

**DOI:** 10.4014/jmb.2110.10039

**Published:** 2022-01-16

**Authors:** Jun-Pil Jang, Mina Jang, Toshihiko Nogawa, Shunji Takahashi, Hiroyuki Osada, Jong Seog Ahn, Sung-Kyun Ko, Jae-Hyuk Jang

**Affiliations:** 1 Anticancer Agent Research Center, Korea Research Institute of Bioscience and Biotechnology (KRIBB), Cheongju 28116, Republic of Korea; 2 Department of Biomolecular Science, KRIBB school of Bioscience, University of Science and Technology (UST), Daejeon 34141, Republic of Korea; 3 RIKEN Center for Sustainable Research Science, 2-1 Hirosawa, Wako, Saitama 351-0198, Japan; 4 Natural Products Biosynthesis Research Unit and RIKEN-KRIBB Joint Research Unit, RIKEN Center for Sustainable Research Science, 2-1 Hirosawa, Wako, Saitama 351-0198, Japan; 5 New Drug Development Center, Daegu-Gyeongbuk Medical Innovation Foundation, Daegu 41061, Republic of Korea

**Keywords:** Oxindole, *Streptomyces* sp., structural elucidation, anti-angiogenic activity

## Abstract

A chemical investigation of a culture extract from *Streptomyces* sp. RK85-270 led to the isolation and characterization of two new oxindoles, RK-270D (1) and E (2). The structures of **1** and **2** were determined by analyzing spectroscopic and spectrometric data from 1D and 2D NMR and High-resolution electrospray ionization mass spectrometry (HRESIMS) experiments. Compound 1 exhibited anti-angiogenic activities against human umbilical vein endothelial cells (HUVECs) without cytotoxicity. Results of Western blot analysis revealed that 1 inhibits VEGF-induced angiogenesis in the HUVECs via VEGFR2/ p38 MAPK-mediated pathway.

## Introduction

Many small molecules derived from natural products have enabled drug discovery. Over the last four decades, 66% of approved small-molecule drugs were naturally derived or inspired by natural products [1]. Particularly, secondary metabolites produced by actinomycetes with their unique structural scaffolds have been prolific chemical sources of bioactive organic compounds [2]. More than half of all naturally derived antibiotics were isolated from actinomycetes, and 70% of these were found in *Streptomyces* [3]. We recently reported novel metabolites isolated from *Streptomyces* species, such as jejucarbazoles A, B, and C [4], aturanosides A and B [5], pentaminomycins A and B [6], and octaminomycins A and B [7].

Cell migration is involved in a number of physiological processes including ovulation, embryonic development, tissue regeneration (wound healing), and inflammation. These migration activities of cells in vitro to be related to many in vivo cellular behaviors such as tumor angiogenesis and metastasis [8]. Then, cell migration is attracting much attention as one of the alternative strategies for development of anti-cancer chemotherapies. Several natural products such as withaferin A [9], prodigiosin [10], and migrastatin [11] have been reported to inhibit cell migration.

We have previously reported the isolation and structural elucidation of three new oxindole derivatives, RK-270A, B, and C from the *Streptomyces* sp. RK85-270 in our search for novel and bioactive secondary metabolites from actinomycetes [12]. Further, an investigation of minor fractions from this fermentation resulted in the isolation of new oxindole derivatives, RK-270D (**1**) and E (**2**). In this study, we describe the isolation, structural elucidation, and anti-angiogenic activity of these compounds.

## Materials and Methods

### General Experimental Procedures

The specific rotations were measured on a JASCO P-1020 polarimeter (JASCO Corporation, Japan) that uses a 100-mm glass microcell. UV spectra were recorded on an Optizen 2120 UV spectrophotometer (Mecasys, Korea). The IR spectra were recorded on a Bruker VERTEX80V FT-IR spectrometer (Bruker, Germany). The NMR spectra were recorded on Bruker AVANCE HD 800 NMR spectrometer (Bruker, USA) at the Korea Basic Science Institute (KBSI) in Ochang, South Korea. Chemical shifts were referenced to a residual solvent signal (DMSO-*d_6_* δ_H_ 2.50, δ_C_ 39.51). High-resolution electrospray ionization mass spectrometry (HRESIMS) data were acquired using Q-TOF mass spectrometer (Waters, USA) on an SYNAPT G2 at KBSI in Ochang, South Korea. Column chromatography was performed on reversed-phase silica gel (0.075 mm; Cosmosil, Japan). Analytical C_18_ (5 μm, 4.6 × 150 mm; Cosmosil, Japan) and semipreparative C_18_ (10 μm, 10 × 250 mm; Cosmosil, Japan) columns were used for reversed-phase HPLC on a YL900 HPLC system (Young Lin, Korea) equipped with a YL9120 UV/vis detector (Young Lin, Korea) that used HPLC grade solvents (Burdick & Jackson, USA). Open column chromatography was performed using a silica gel (silica gel 60 (0.063-0.200 mm); Merck, USA). Vacuum liquid column chromatography was conducted with an ODS (Cosmosil, 75 μm). Semi preparative C_18_ (Cosmosil 5C_18_-MS-II, 5 μm, 10 × 250 mm) columns were used for HPLC on a YL9100 HPLC system equipped with a photodiode array detector (YL9160) that uses HPLC grade solvents.

### Fermentation and Isolation of Compounds

RK85-270 was cultured in a medium consisting of soluble starch (10 g), yeast extract (1 g), NZ-amine (1 g), and agar (15 g) in 1.0 L of distilled water at pH 7.0. The stock culture was cultured in a 250 ml Erlenmeyer flask containing 50 ml of seed culture medium (soluble starch 1%, yeast extract 0.1%, and tryptone 0.1%) for 3 days at 28°C on a rotary shaker with agitated at 165 rpm [12]. For a large culture (10 L), 1% of the preculture broth was inoculated into 40 × 1,000 ml baffled Erlenmeyer flasks containing 250 ml of modified CDY broth (glucose 2%, soluble starch 1%, meat extract 0.3%, yeast extract 0.25%, K_2_HPO_4_ 0.005%, NaCl 0.05%, CaCO_3_ 0.05%, and MgSO_4_·7H_2_O 0.05%), which was cultured for 8 days at 28°C on a rotary shaker agitated at 165 rpm. The mixture was then centrifuged, and the supernatant was extracted with EtOAc, while the mycelium was extracted with acetone. After concentrating the residual solvents under reduced pressure, the two portions were combined and dried to yield an extract of 1.1 g of the *Streptomyces* sp. RK85-270 of extract. The dried extract (1.1 g) was then fractionated by reversed phase C_18_ vacuum column chromatography with a stepwise solvent system of MeOH-H_2_O (20:80 to 100:0, each × 1 L) to yield nine fractions. The compounds **1** (2.4 mg, *t*_R_ 25.9 min) and **2** (2.8 mg, *t*_R_ 28.6 min) were obtained when the fraction was eluted with MeOH-H_2_O (60:40, 65.8 mg) and purified via reversed phase HPLC (Cosmosil, semipreparative C_18_, 45% CH_3_CN, 3 mL/min, UV detection at 210 and 270 nm).

### Characterization of Compounds

RK-270D (**1**): a yellowish powder; ${\left[\text{α}\right]}_{\text{D}}^{\text{25}}$- 0.5 (c 0.05, MeOH); IR (ATR) *ν*_max_ (cm^-1^) 3400, 3200, 2917, 1683, 1614, 1556; UV (MeOH) λ_max_ (log ε) 228 (3.37), 258 (4.20) 264 (4.11), 297 (3.55); ^1^H and ^13^C NMR data, Table 1; HRESIMS *m/z* 293.1268 [M + Na]^+^ (calcd for C_16_H_18_N_2_O_2_Na, 293.1266).

RK-270E (**2**): a yellowish powder; ${\left[\text{α}\right]}_{\text{D}}^{\text{25}}$- 16 (c 0.05, MeOH); IR (ATR) *ν*_max_ (cm^-1^) 3385, 2900, 1683, 1622, 1446, 1380; UV (MeOH) λ_max_ (log ε) 230 (3.37), 258 (4.20) 264 (4.11), 297 (3.55); ^1^H and ^13^C NMR data, Table 1; HRESIMS *m/z* 295.1427 [M + Na]^+^ (calcd for C_16_H_20_N_2_O_2_Na, 295.1422).

### Cell Viability Assay

HUVECs were cultured in EGM-2 bulletkit medium (Lonza, CC-3156 & CC-4176) supplemented with 10%fetal bovine serum (Welgene, S001-07), 100 units/mL penicillin, and 100 μg/ml streptomycin (Gibco, 15140-122). HUVECs were seeded in 96-well cell culture plates (1 × 10^4^ cells/well) and treated with various concentrations of **1** or **2** for 24 h. The cell viability was measured using the EZ-Cytox colorimetric assay (Daeil Lab service, 0793) according to the manufacturer’s protocol.

### Wound Healing Assay

HUVECs were seeded in a confluent monolayer in 24-well cell culture plates (6 × 10^4^ cells/well). Cells were scratched with a yellow tip, washed with PBS to remove nonadherent cells, and then treated with 1 μg/ml mitomycin C (Roche, 10107409001) for 3 h. After incubation, cells were treated with **1** or **2** for 12 h and fixed in 4%paraformaldehyde for 10 min. Cells stained with 0.2% crystal violet were observed under a microscope at 100× magnification.

### Transwell Chamber Invasion Assay

The cell invasion assay was performed using 6.5 mm transwells with 8.0 μm pore polycarbonate membrane inserts (Corning, 3422). Starved HUVECs (1 × 10^5^ cells/well) with EBM-2 basal medium without serum and growth factors were seeded in the Matrigel-coated upper chamber with **1** or **2** in basal medium for 24 h, whereas EGM-2 medium containing VEGF (30 ng/ml) (Sigma, V7259) was loaded in the lower chamber. The invasive cells were fixed in 4% paraformaldehyde for 10 min, stained with 0.2% crystal violet, and observed under a microscope at 100× magnification.

### Capillary Tube Formation Assay

Starved HUVECs (2 × 10^4^ cells/well) were treated with **1** or **2** in the presence of VEGF (30 ng/ml) in Matrigel (Corning, 356234)-coated 96-well cell culture plates. After 3 h incubation, the tubular capillary structures were captured using a microscope at 100× magnification.

### Western Blot Analysis

Starved HUVECs were incubated with **1** for 12 h, and then treated with VEGF (30 ng/ml) for 5 min. Cells were directly lysed with the 2× SDS sample buffer, and the proteins were separated via SDS-PAGE. The membranes were blocked with 5% skim milk and probed with the indicated primary antibodies at 4°C overnight, after transferring onto nitrocellulose membranes (0.2 μm; Bio-Rad) of the proteins. Primary antibodies against VEGF receptor 2 (#2479), p-VEGF receptor 2 (Y1175, #2478), p38 (#9212), and p-p38 (Thr180/Tyr182, #4631) were purchased from Cell Signaling Technology. A primary antibody against β-actin (sc-47778) was obtained from Santa Cruz Biotechnology. After incubation for 1 h with horseradish peroxidase-conjugated secondary anti-rabbit or anti-mouse antibodies (Cell Signaling Technology, #7074 / #7076), protein bands were detected using the SuperSignal West Pico chemiluminescent substrate or SuperSignal West Femto maximum sensitivity substrate (ThermoFisher Scientific, #34080 / #34095).

## Results and Disscussion

### Structural Determination of Compounds

**Fig. 1:**
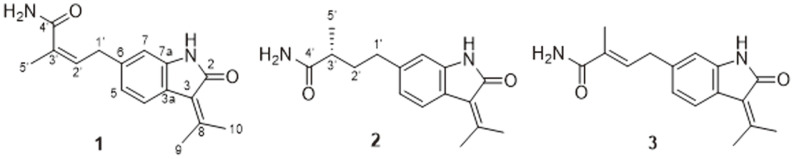
Structures of compounds 1−3.

**Fig. 2:**
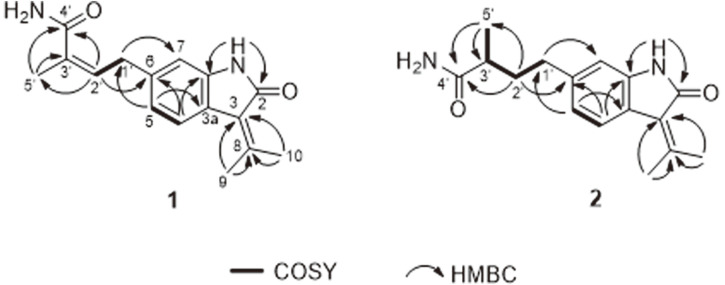
Key 2D NMR correlations of compounds **1** and **2**.

Compound **1** (**1**) was isolated as a yellowish powder and its molecular formula was established as C_16_H_18_N_2_O_2_ based on HRESIMS analysis. The characteristic UV absorptions at 206, 254, and 265 nm suggested the presence of an indole chromophore in **1**. The ^1^H NMR spectrum in DMSO-*d_6_* showed resonances for three methyls (δ_H_ 1.85, s; 2.29, s; and 2.49, s), an olefinic multiplet (δ_H_ 5.26, m), and a methylene (δ_H_ 3.48, d, *J* = 7.3 Hz). It also showed an exchangeable NH proton (δ_H_ 10.35, s) and an aromatic ABX spin system for a 1,2,4-trisubstituted aromatic ring at δ_H_ 7.42 (d, *J* = 8.0 Hz), 6.80 (dd, *J* = 8.0, 1.2 Hz), and 6.68 (d, *J* = 1.2 Hz). The ^13^C NMR data of **1** (Table 1) showed 16 carbon resonances assigned by HSQC-DEPT data, which can be attributed to three methyls, one methylene, four methines, six quaternary carbons, and two carbonyls. The NMR data of **1** were almost similar to those of RK-270C (3), a known oxindole metabolite from *Streptomyces* sp. RK85-270. Small differences were observed in the ^13^C NMR chemical shifts for C-5′ (δ_c_ 21.2 in **1**; δ_c_ 13.3 in RK-270C) [12]. The planar structure of **1** was supported by interpretation of COSY and HMBC spectra (Fig. 2). The geometry of Δ^2^ was assigned as Z based on the ^13^C NMR chemical shift value of Me-5′ at 21.2 ppm. Therefore, **1** was a new geometric isomer of RK-270C at Δ^3^′, namely, RK-270D (Fig. 1).

Compound **1** (**2**) was obtained as a yellowish powder. The molecular formula C_16_H_20_N_2_O_2_ of **2** was determined by HRESIMS. The difference of two mass units compared to **1** (C_16_H_18_N_2_O_2_) indicated saturation in one of the double bonds. The ^1^H and ^13^C NMR resonances were similar to those of **1** except for the resonances of a methine proton at δ_H_ 2.24 (1H, dd, *J* = 7.3 Hz, H-3′) and a methylene protons at δ_H_ 1.78, 1.51 (2H, m, H-2′). The NMR data of **2** (Table 1) were similar to those of 1. The main difference was the presence of a methine (δ_H_ 2.24, dd, H-3′; *J* = 7.3 Hz). The structure of **2** was determined to be a saturated analogue of **1**. The proposed structure was fully supported by 2D NMR experiments (Fig. 2). The absolute configuration at C-3′ as determined by the negative optical value of **2** (${\left[\text{α}\right]}_{\text{D}}^{\text{25}}$-16) in comparison with the literature [13]. This established that the C-3′ position in **2** was supposed to be R-configuration, and the structure of **2** was designated as RK-270E.

### RK-270D (1) Inhibited the Migration, Invasion, and Capillary Tube Formation of Human Umbilical Vein Endothelial Cells (HUVECs)

**Fig. 3:**
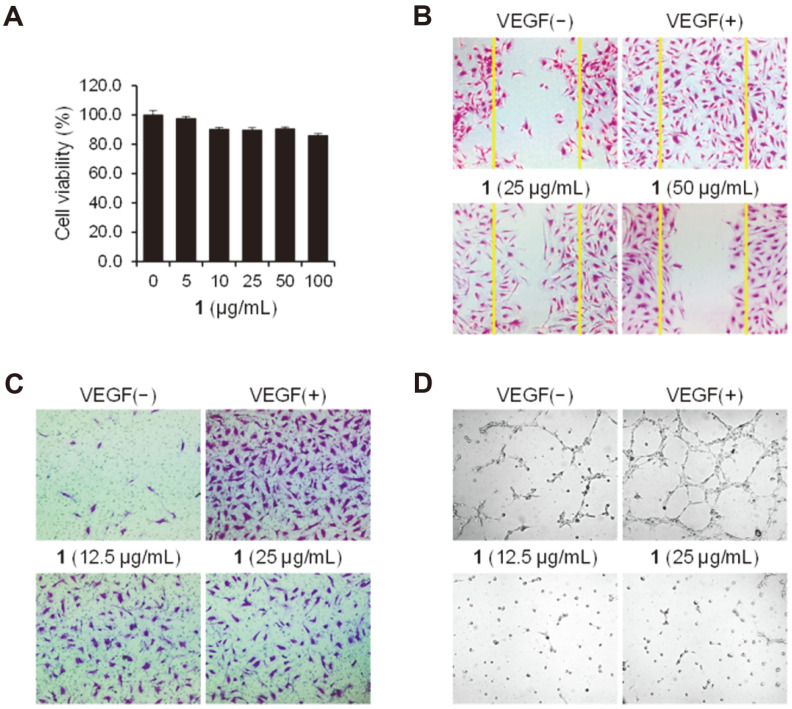
Anti-angiogenic activity of compound 1 in HUVECs. (**A**) Effect of **1** on cell viability. HUVECs were treated with the indicated concentrations of **1** for 24 h. Cell viability was measured using the EZ-Cytox assay solution (mean ± SD, *n* = 3). (**B**−**D**) Representative images of the inhibitory effect of **1** on cell migration (**B**), invasion (**C**), and capillary tube formation (**D**). (**B**) Cells were treated with the indicated concentrations of **1** in the presence of VEGF (30 ng/ml) for 12 h and stained with crystal violet. The migrated cells were observed under a microscope. Cells not stimulated with VEGF were used as a negative control. (**C**) Starved cells were treated with **1** and VEGF (30 ng/ml) in the Matrigel-coated upper chamber. After 18 h incubation, the invaded cells were stained with crystal violet and imaged by a microscope. (**D**) Starved cells were treated with **1** and VEGF (30 ng/ml) for 3 h in the Matrigel-coated 96-well cell culture plates. Capillary-like tubular structures were captured under a microscope.

Angiogenesis occurs sequentially in the following processes, including endothelial cell proliferation, sprouting cell migration, and invasion to generate new blood vessels [14]. Angiogenesis inhibitors are being used clinically to treat cancer, macular degeneration in the eye, and other diseases [15, 16]. The cell viability was tested in HUVECs before evaluating the anti-angiogenic effects of **1** and **2**. Compounds **1** and **2** did not show cytotoxicity against HUVECs up to the concentration of 100 μg/ml (Figs. 3A and S13A). We first tested cell migration and invasion of HUVECs through wound healing and Matrigel invasion assays, respectively, to investigate the angiogenic effects of **1** (Figs. 3B and 3C). As shown in Figs. 3B and 3C, **1** inhibited cell migration and vascular endothelial growth factor (VEGF)-induced invasion of HUVECs in a dose-dependent manner. Further, we performed a capillary tube formation assay to investigate phenotypic effects of **1** as angiogenesis inhibitors (Fig. 3D). The tubular capillary structures on the Matrigel were effectively generated in HUVECs treated with VEGF, a signal protein that stimulates the formation of blood vessels. In this assay system, the potent anti-angiogenic effects were observed in the HUVECs treated with **1**. On the contrary the anti-angiogenic activity of **2** was not observed (Figs. S13B−D). Our results suggest that **1** inhibits the VEGF-induced angiogenesis of HUVECs in vitro.

### RK-270D (1) Inhibits the VEGF-Induced Angiogenesis of HUVECs via VEGFR2-Mediated Signaling

**Fig. 4:**
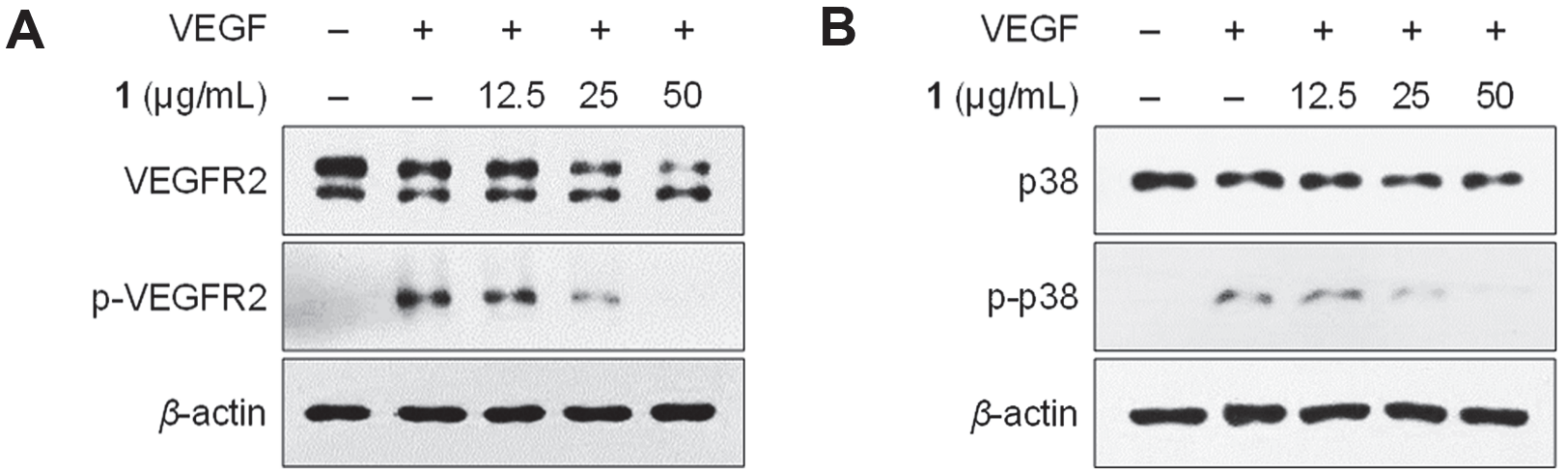
Effects of compound 1 on the phosphorylation of VEGFR2 and p38 in VEGF-induced HUVECs. (**A**, **B**) HUVECs were pretreated with **1** followed by stimulation with VEGF (30 ng/ml) for 5 min. Phosphorylation of VEGFR2 (**A**) and p38 MAPK (**B**) was analyzed via Western blot analysis using antibodies against VEGFR2, p-VEGFR2, p38, and p-p38. Cells not stimulated with VEGF were used as a negative control.

VEGF receptor 2 (VEGFR2), a main VEGF receptor protein, is a key regulator of angiogenesis [17]. HUVECs were preincubated with **1** and treated with VEGF immediately to determine whether **1** inhibits the VEGF-induced angiogenesis via the VEGFR2-mediated signaling pathway. As expected, Western blot analysis showed that **1** efficiently inhibited the phosphorylation of VEGFR2 (Fig. 4A). VEGFR2 signaling has been reported to drive activation of ERK and p38 mitogen-activated protein kinases (MAP kinases) [18]. The phosphorylation of p38 was decreased in HUVECs treated with **1**, whereas the decrease in phosphorylation of ERK was not observed (Fig. 4B, S14). These data suggest that **1** inhibits the VEGF-induced angiogenesis via VEGFR2/p38 pathway in HUVECs.

## Supplemental Materials

Supplementary data for this paper are available on-line only at http://jmb.or.kr.

## Figures and Tables

**Table 1:** NMR data for compounds **1** and **2** in DMSO-*d_6_*.

Position	1		2	

	δ_C_	δ_H_ (*J* in Hz)	δ_C_	δ_H_ (*J* in Hz)
1-NH		10.36, s		10.35, s
2	169.3		169.3	
3	123.1		123.2	
3a	122.1		121.9	
4	124.0	7.42, d (8.0)	124.0	7.42, d (8.0)
5	121.5	6.80, dd (8.0, 1.2)	121.3	6.77, dd (8.0, 1.2)
6	140.7		142.4	
7	109.6	6.68, d (1.2)	109.6	6.63, d (1.2)
7a	141.1		141.0	
8	153.1		153.0	
9	25.2	2.29, s	25.2	2.29, s
10	22.6	2.49, s	22.5	2.49, s
1′	35.4	3.48, d (7.3)	33.6	2.48, overlapped
2′	130.5	5.52, td (7.3, 1.2)	35.8	1.78, m
				1.51, m
3′	133.3		39.5	2.24, dd (14.4, 6.8)
4′	176.4		178.0	
5′	21.2	1.85, s	18.4	1.03, d (6.8)
4′-NH_2_		7.15, brs		6.73, brs
		7.35, brs		7.26, brs

^1^H and ^13^C data were recorded at 800 and 200 MHz, respectively.

## References

[1] Newman DJ, Cragg GM (2020). Natural products as sources of new drugs over the nearly four decades from 01/1981 to 09/2019. J. Nat. Prod..

[2] Fenical W, Jensen PR (2006). Developing a new resource for drug discovery: marine actinomycete bacteria. Nat. Chem. Biol..

[3] Saito S, Kato W, Ikeda H, Katsuyama Y, Ohnishi Y, Imoto M (2020). Discovery of "heat shock metabolites" produced by thermotolerant actinomycetes in high-temperature culture. J. Antibiot..

[4] Kim GS, Jang J-P, Kwon M, Oh TH, Heo KT, Lee B (2021). Jejucarbazoles A-C, carbazole glycosides with indoleamine 2,3-dioxygenase 1 inhibitory activity from *Streptomyces* sp. KCB15JA151. RSC. Adv..

[5] Jang J-P, Hwang GJ, Jang M, Takahashi S, Ko SK, Osada H (2018). Aturanosides A and B, glycosylated anthraquinones with antiangiogenic activity from a soil-derived *Streptomyces* species. J. Nat. Prod..

[6] Jang J-P, Hwang GJ, Kwon MC, Ryoo IJ, Jang M, Takahashi S (2018). Pentaminomycins A and B, hydroxyarginine-containing cyclic pentapeptides from *Streptomyces* sp. RK88-1441. J. Nat. Prod..

[7] Jang J-P, Nogawa T, Futamura Y, Shimizu T, Hashizume D, Takahashi S (2017). Octaminomycins A and B, cyclic octadepsipeptides active against *Plasmodium falciparum*. J. Nat. Prod..

[8] Carmeliet P (2003). Angiogenesis in health and disease. Nat. Med..

[9] Lee J, Hahm E-R, Singh SV (2010). Withaferin A inhibits activation of signal transducer and activator of transcription 3 in human breast cancer cells. Carcinogenesis.

[10] Zhang J, Shen Y, Liu J, Wei D (2005). Antimetastatic effect of prodigiosin through inhibition of tumor invasion. Biochem. Pharmacol..

[11] Nakae K, Yoshimoto Y, Sawa T, Homma Y, Hamada M, Takeuchi T (2005). Migrastatin, a new inhibitor of tumor cell migration from *Streptomyces* sp. MK929-43F1. Taxonomy, fermentation, isolation and biological activities. J. Antibiot..

[12] Jang J-P, Nogawa T, Uramoto M, Okano A, Futamura Y, Shimizu T (2015). RK-270A-C, new oxindole derivatives isolated from a microbial metabolites fraction library of *Streptomyces* sp. RK85-270. J. Antibiot..

[13] Zheng D, Han L, Jiang Y, Cao YR, Liu J, Chen X (2013). Structure elucidation of four prenylindole derivatives from *Streptomyces* sp. isolated from *Ailuropoda melanoleuca* feces. Magn. Reson. Chem..

[14] Bryan BA, D'Amore PA (2007). What tangled webs they weave: Rho-GTPase control of angiogenesis. Cell. Mol. Life. Sci..

[15] Rini BI (2007). Vascular endothelial growth factor-targeted therapy in renal cell carcinoma: current status and future directions. Clin. Cancer Res..

[16] Ng EWM, Adamis AP (2005). Targeting angiogenesis, the underlying disorder in neovascular age-related macular degeneration. Can. J. Ophthalmol..

[17] Olsson AK, Dimberg A, Kreuger J, Claesson-Welsh L (2006). VEGF receptor signalling - in control of vascular function. Nat. Rev. Mol. Cell. Biol..

[18] Lamalice L, Houle F, Jourdan G, Huot J (2004). Phosphorylation of tyrosine 1214 on VEGFR2 is required for VEGF-induced activation of Cdc42 upstream of SAPK2/p38. Oncogene.

