# Supplementary material for: RK-270D and E, Oxindole Derivatives from Streptomyces sp. with Anti-Angiogenic Activity
Source: J Microbiol Biotechnol. 2022 Jan 16;32(3):302–6. doi: 10.4014/jmb.2110.10039 (PMC9628841; doi:10.4014/jmb.2110.10039)
Supplement: Supplementary file 1 [file jmb-32-3-302-supple.pdf]

SUPPLEMENTARY INFORMATION

RK-270D and E, oxindole derivatives from *Streptomyces* sp. with anti-angiogenic activity

Jun-Pil Jang[1,†], Mina Jang[1,†], Toshihiko Nogawa[3], Shunji Takahashi[4], Hiroyuki Osada[3], Jong Seog Ahn[1,2*], Sung-Kyun Ko[1,2*], and Jae-Hyuk Jang[1,2*]

[1] Anticancer Agent Research Center, Korea Research Institute of Bioscience and Biotechnology (KRIBB), Cheongju 28116, Korea

[2] Department of Biomolecular Science, KRIBB school of Bioscience, University of Science and Technology (UST), Daejeon 34141, Korea

[3] RIKEN Center for Sustainable Research Science, 2-1 Hirosawa, Wako, Saitama 351-0198, Japan

[4] Natural Products Biosynthesis Research Unit and RIKEN-KRIBB Joint Research Unit, RIKEN Center for Sustainable Research Science, 2-1 Hirosawa, Wako, Saitama 351-0198, Japan

*Correspondence: jangjh@kribb.re.kr (J.-H.J.); Tel.: +82-43-240-6164; Fax: +82-43-240-6169, ksk1230@kribb.re.kr (S.-K. K.); Tel: +82-43-240-6146; Fax: +82-43-240-6169, jsahn@kribb.re.kr (J.S.A.); Tel.: +82-43-240-6160; Fax: +82-43-240-6169

CONTENTS

Figure S1. ^{1}H NMR spectrum (800 MHz) of RK-270D (**1**) in DMSO-d_6 /page 3

Figure S2. ^{13}C NMR spectrum (200 MHz) of RK-270D (**1**) in DMSO-d_6 /page 3

Figure S3. HSQC-DEPT spectrum of RK-270D (**1**) in DMSO-d_6 /page 4

Figure S4. COSY spectrum of RK-270D (**1**) in DMSO-d_6 /page 4

Figure S5. HMBC spectrum of RK-270D (**1**) in DMSO-d_6 /page 5

Figure S6. HRESIMS spectrum of RK-270D (**1**) /page 5

Figure S7. ^{1}H NMR spectrum (800 MHz) of RK-270E (**1**) in DMSO-d_6 /page 6

Figure S8. ^{13}C NMR spectrum (200 MHz) of RK-270E (**1**) in DMSO-d_6 /page 6

Figure S9. HSQC-DEPT spectrum of RK-270E (**1**) in DMSO-d_6 /page 7

Figure S10. COSY spectrum of RK-270E (**1**) in DMSO-d_6 /page 7

Figure S11. HMBC spectrum of RK-270E (**1**) in DMSO-d_6 /page 8

Figure S12. HRESIMS spectrum of RK-270E (**1**) /page 8

Figure S13. Effects of compound **2** on angiogenesis in HUVECs /page 9

Figure S14. Effects of compound **1** on the phosphorylation of ERK in VEGF-induced HUVECs /page 10

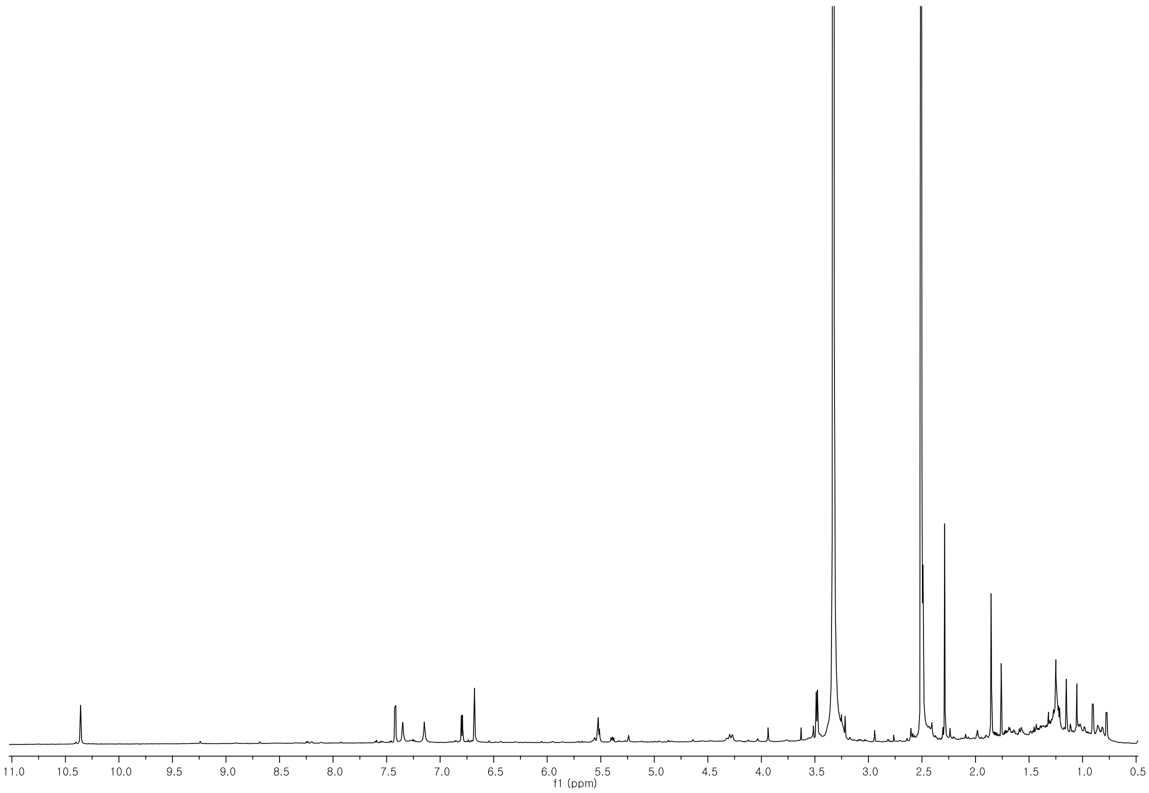

Figure S1. ^{1}H NMR spectrum (800 MHz) of RK-270D (**1**) in DMSO-d_6

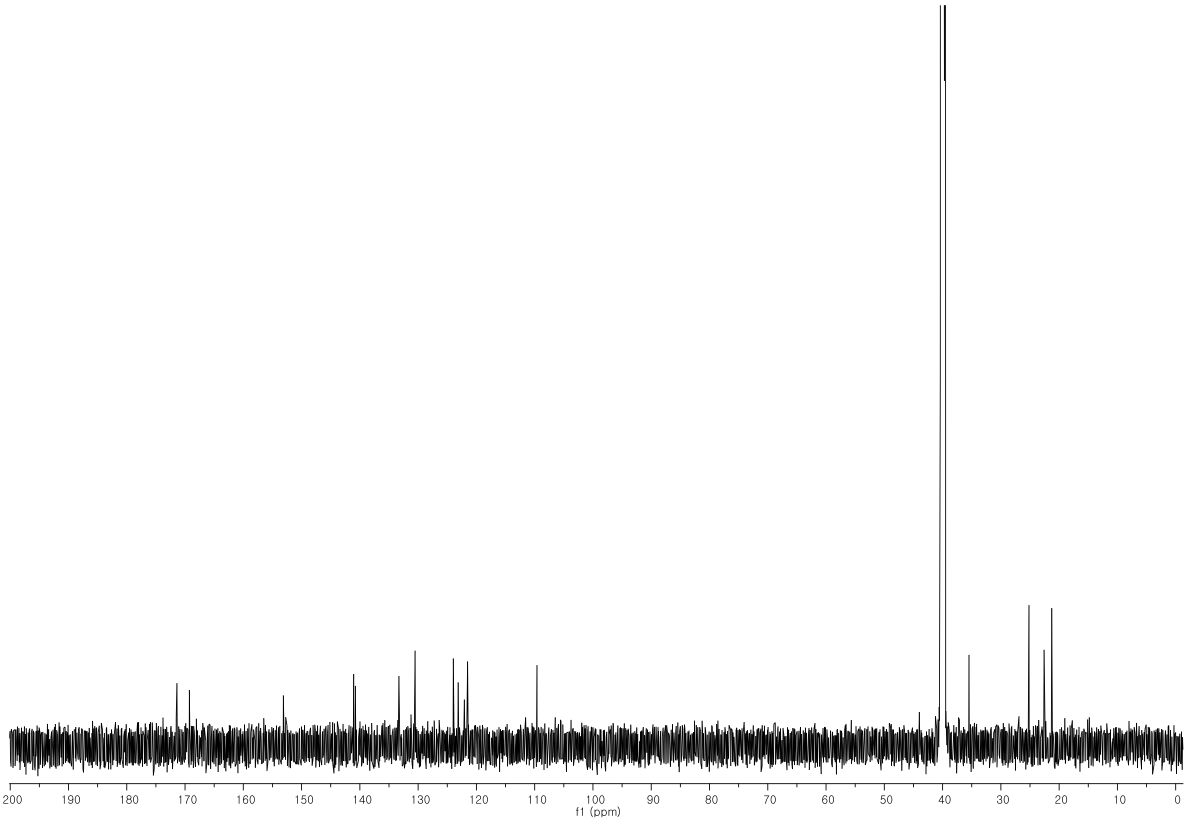

Figure S2. ^{13}C NMR spectrum (200 MHz) of RK-270D (**1**) in DMSO-d_6

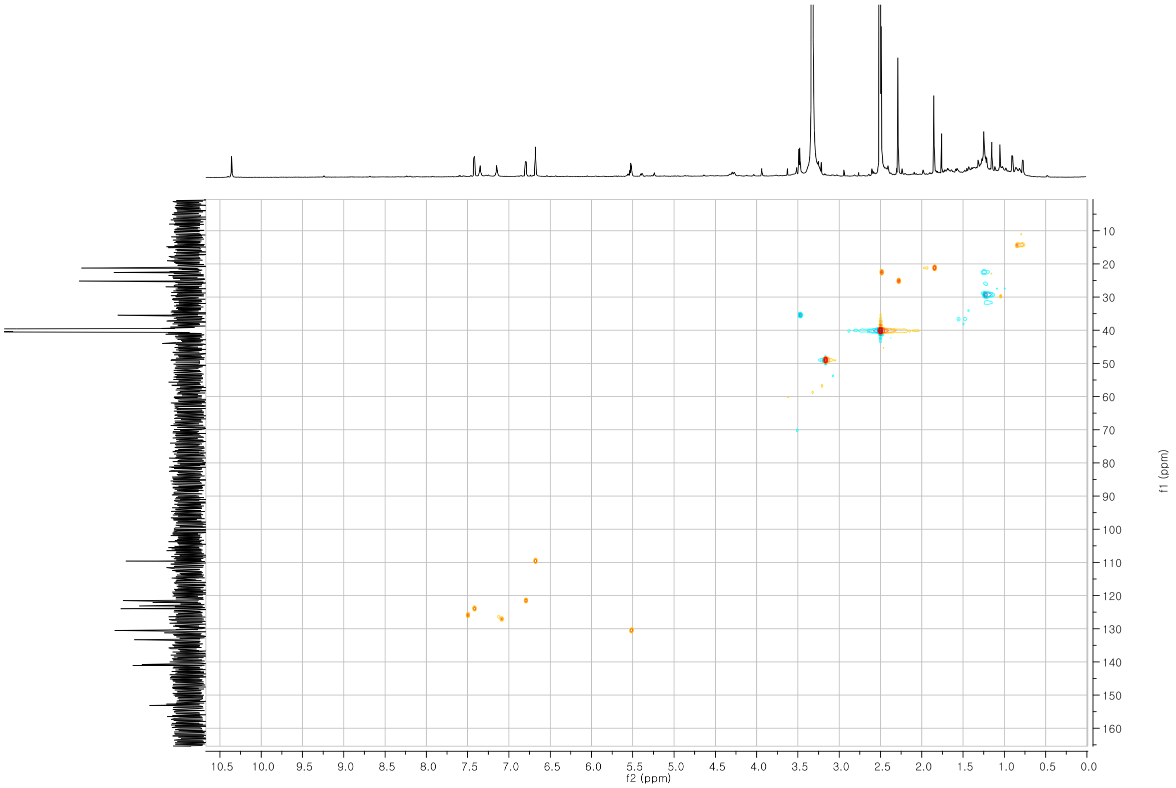

Figure S3. HSQC-DEPT spectrum of RK-270D (**1**) in DMSO-d_6

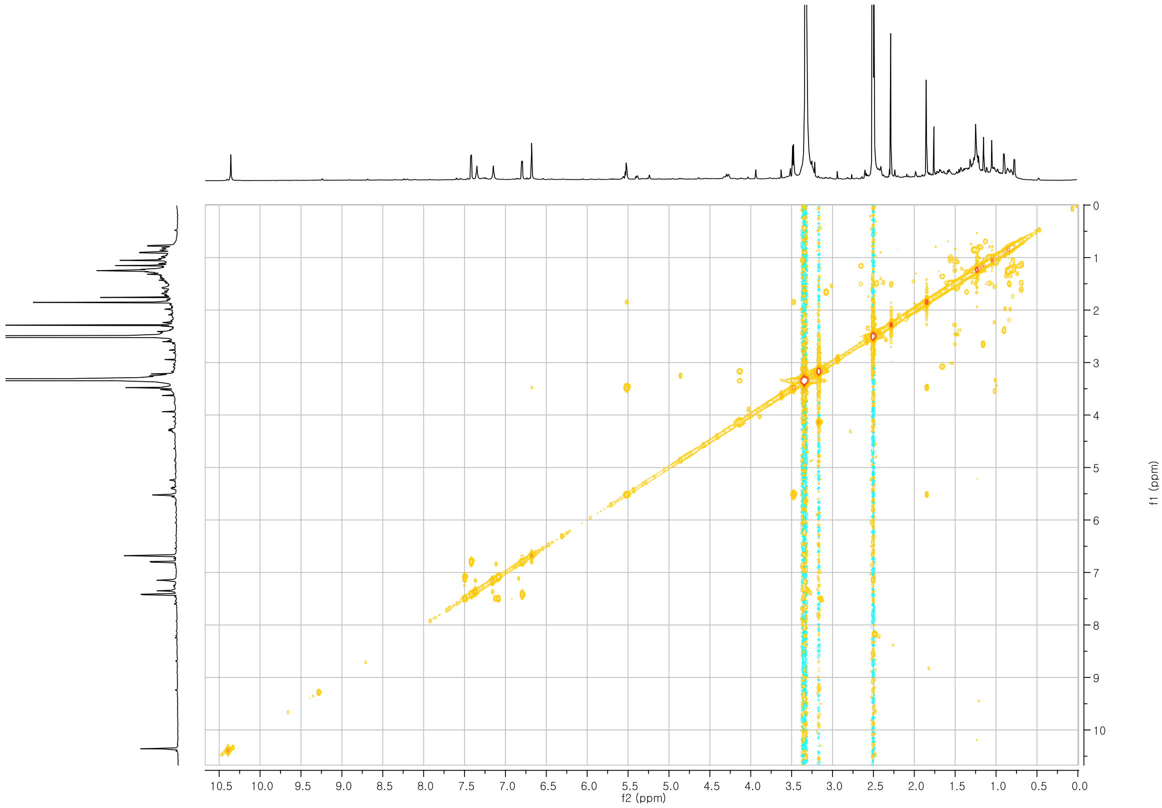

Figure S4. COSY spectrum of RK-270D (**1**) in DMSO-d_6

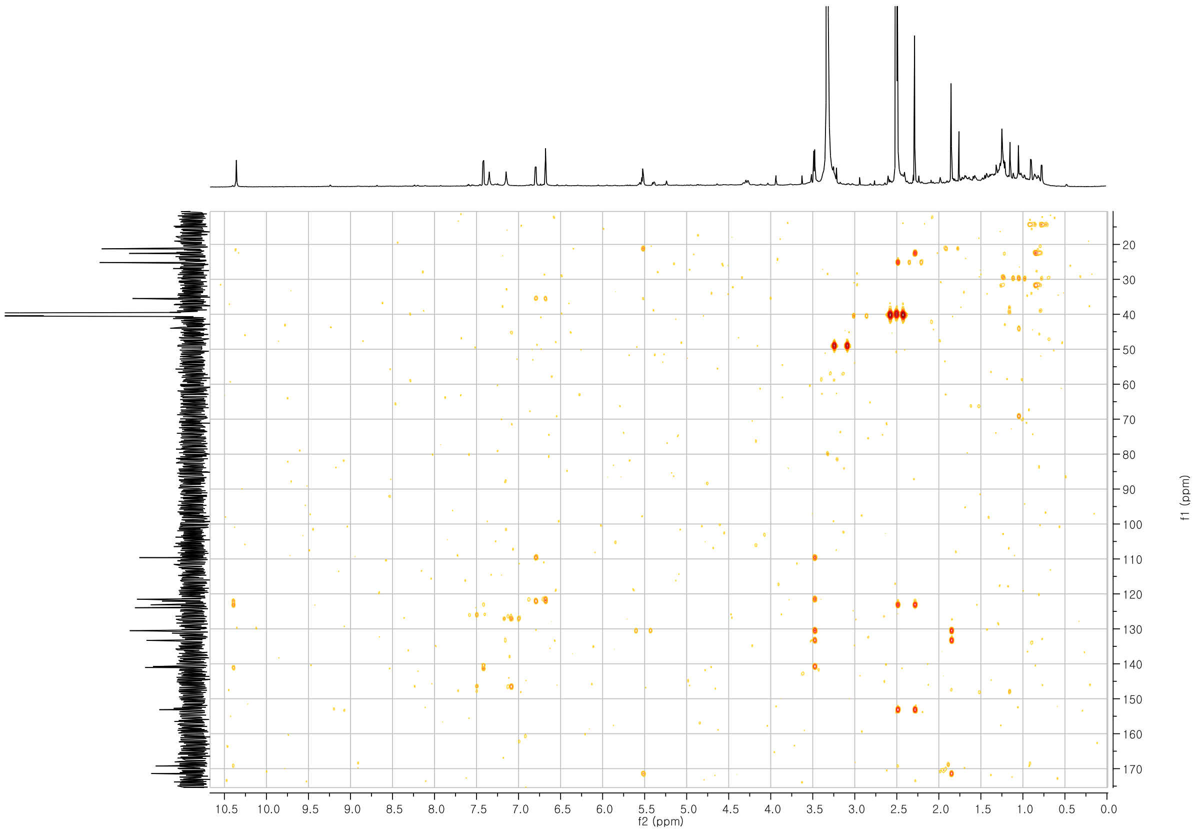

Figure S5. HMBC spectrum of RK-270D (**1**) in DMSO-d_6

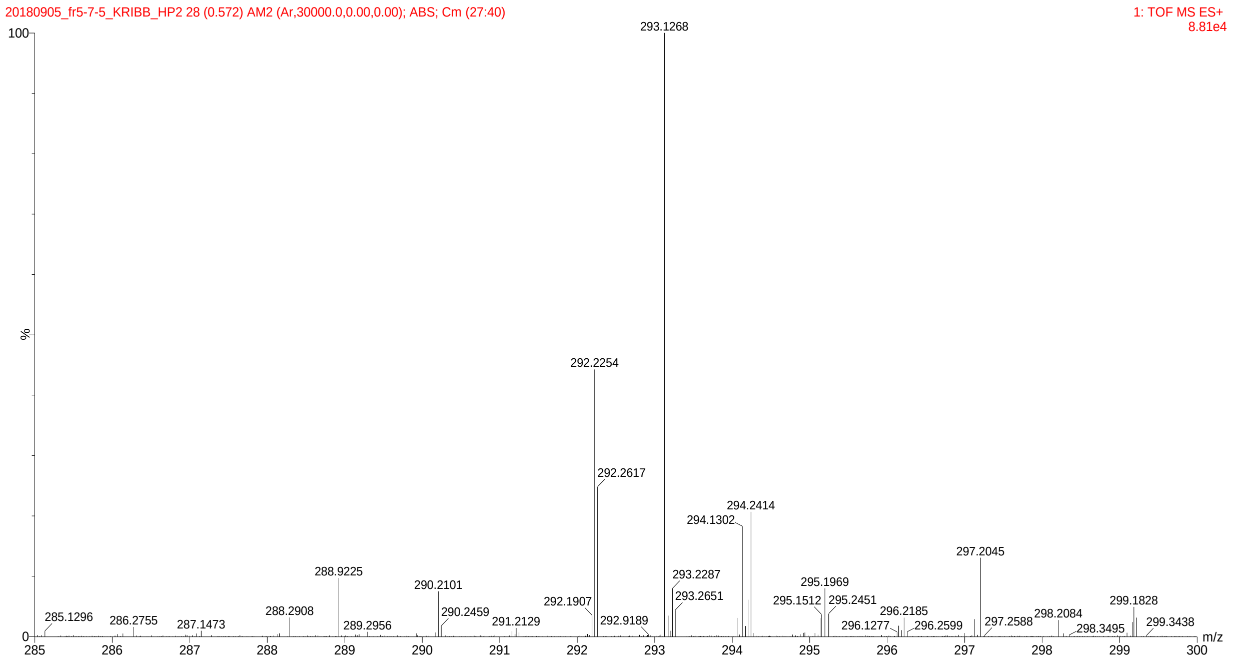

Figure S6. HRESIMS spectrum of RK-270D (**1**)

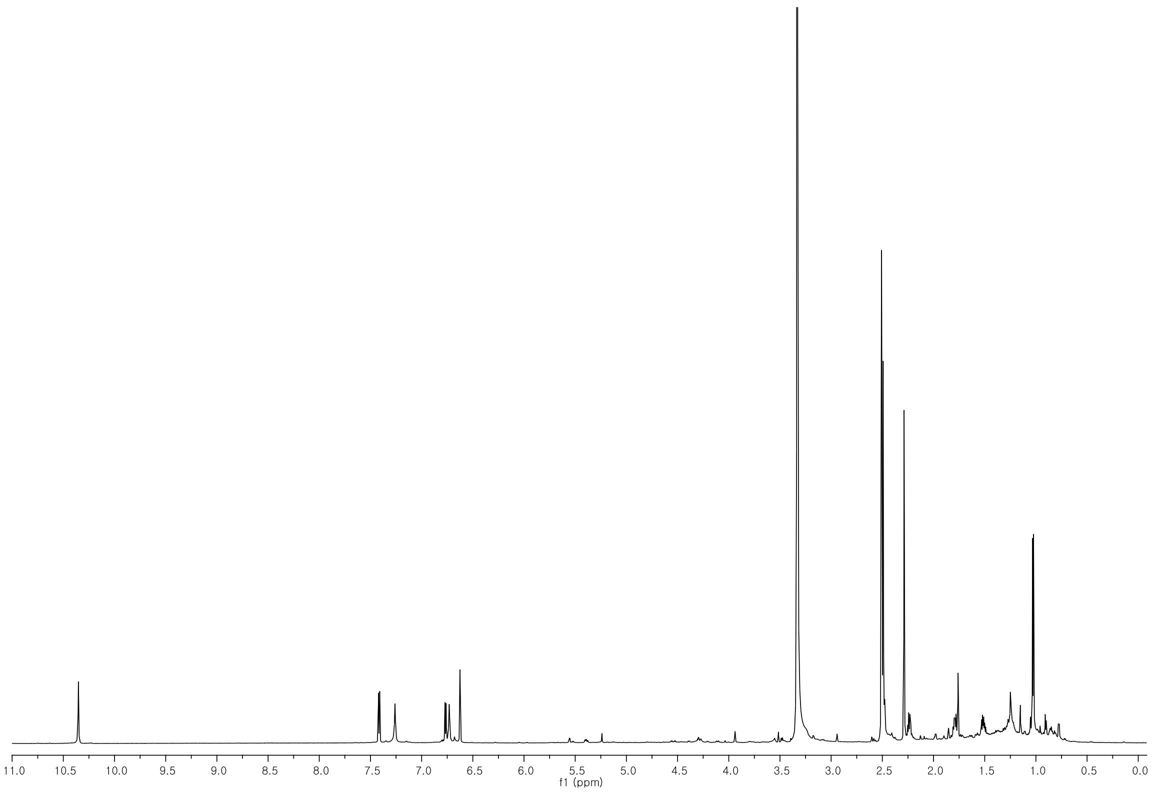

Figure S7. ^{1}H NMR spectrum (800 MHz) of RK-270E (**2**) in DMSO-d_6

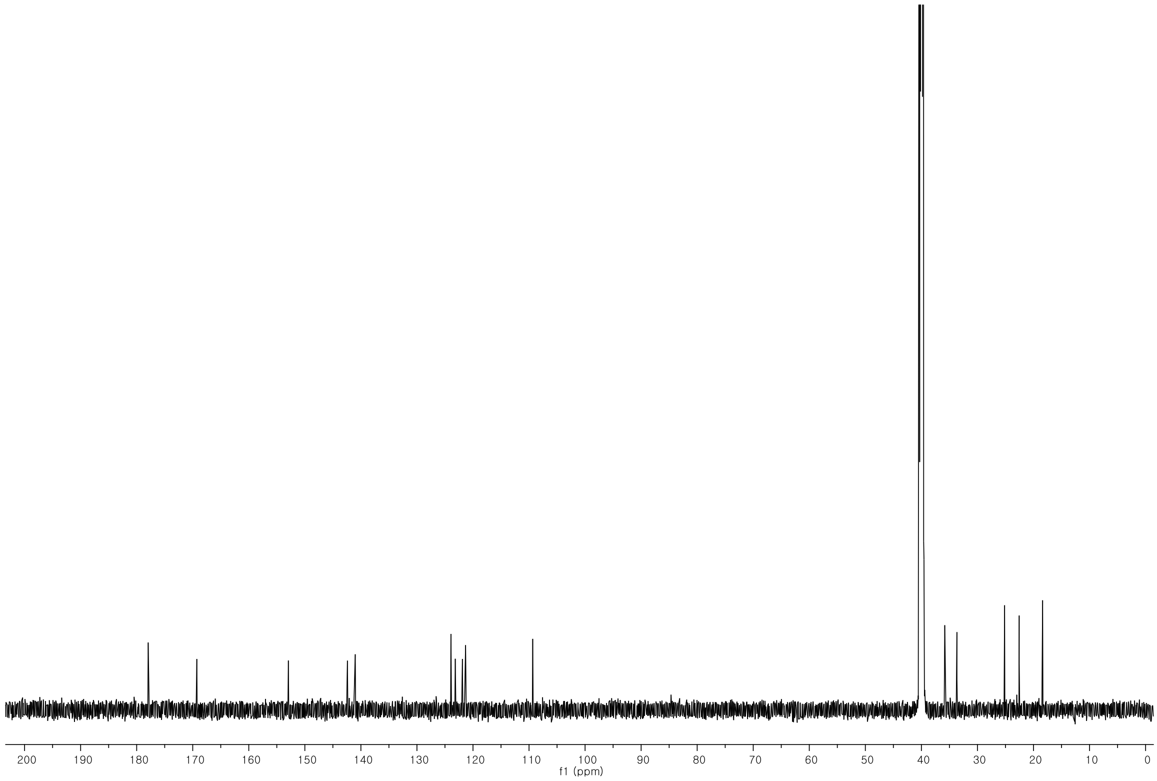

Figure S8. ^{13}C NMR spectrum (200 MHz) of RK-270E (**2**) in DMSO-d_6

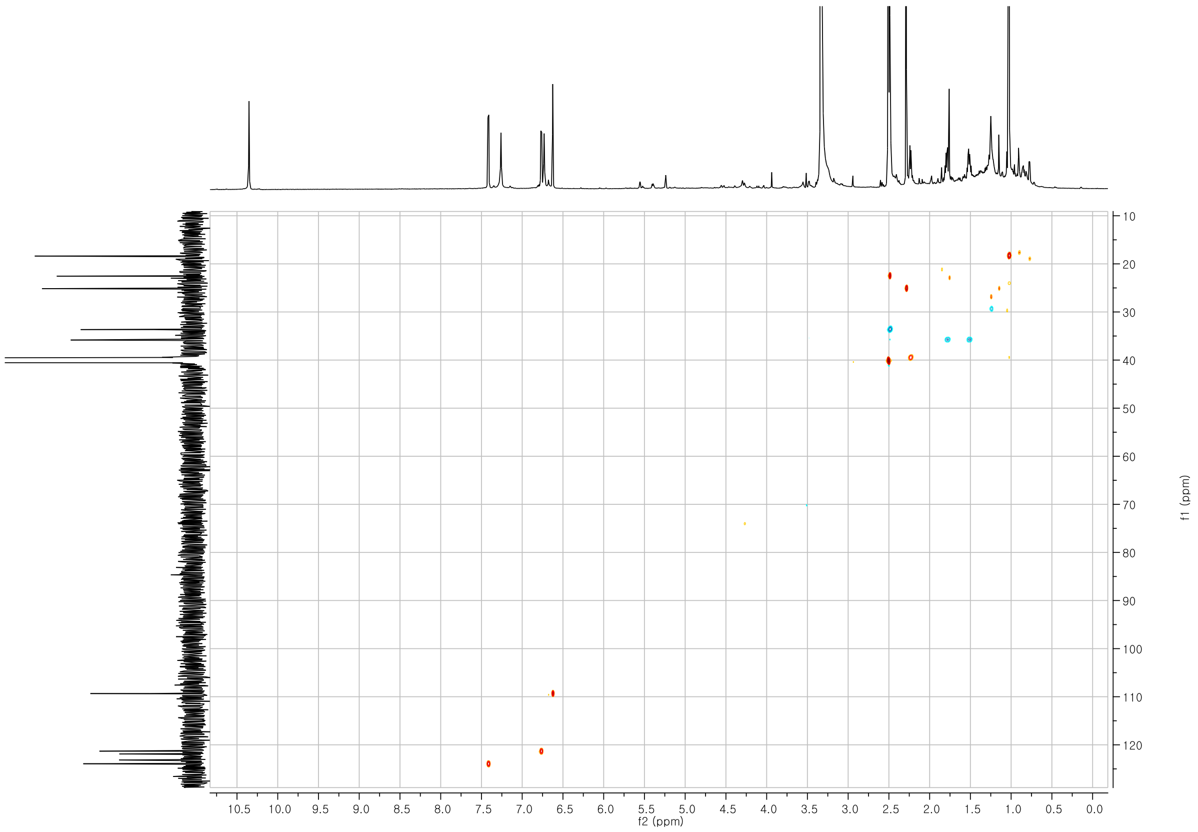

Figure S9. HSQC-DEPT spectrum of RK-270E (**2**) in DMSO-d_6

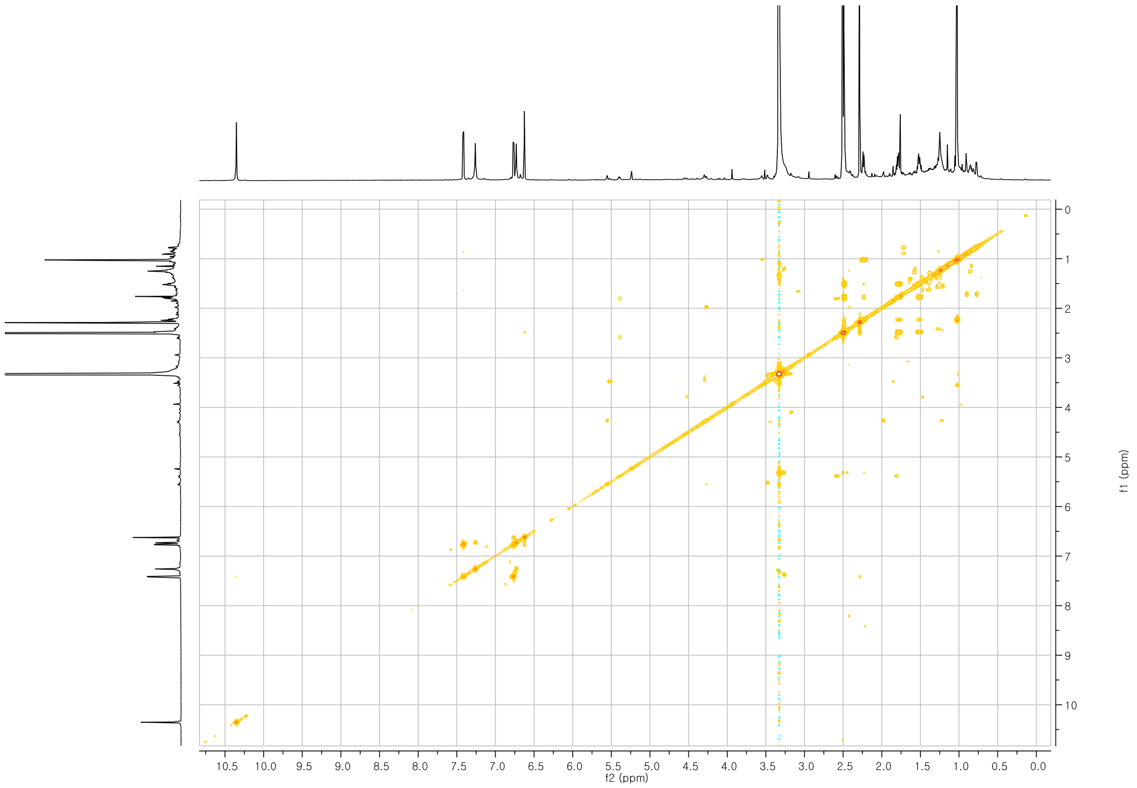

Figure S10. COSY spectrum of RK-270E (**2**) in DMSO-d_6

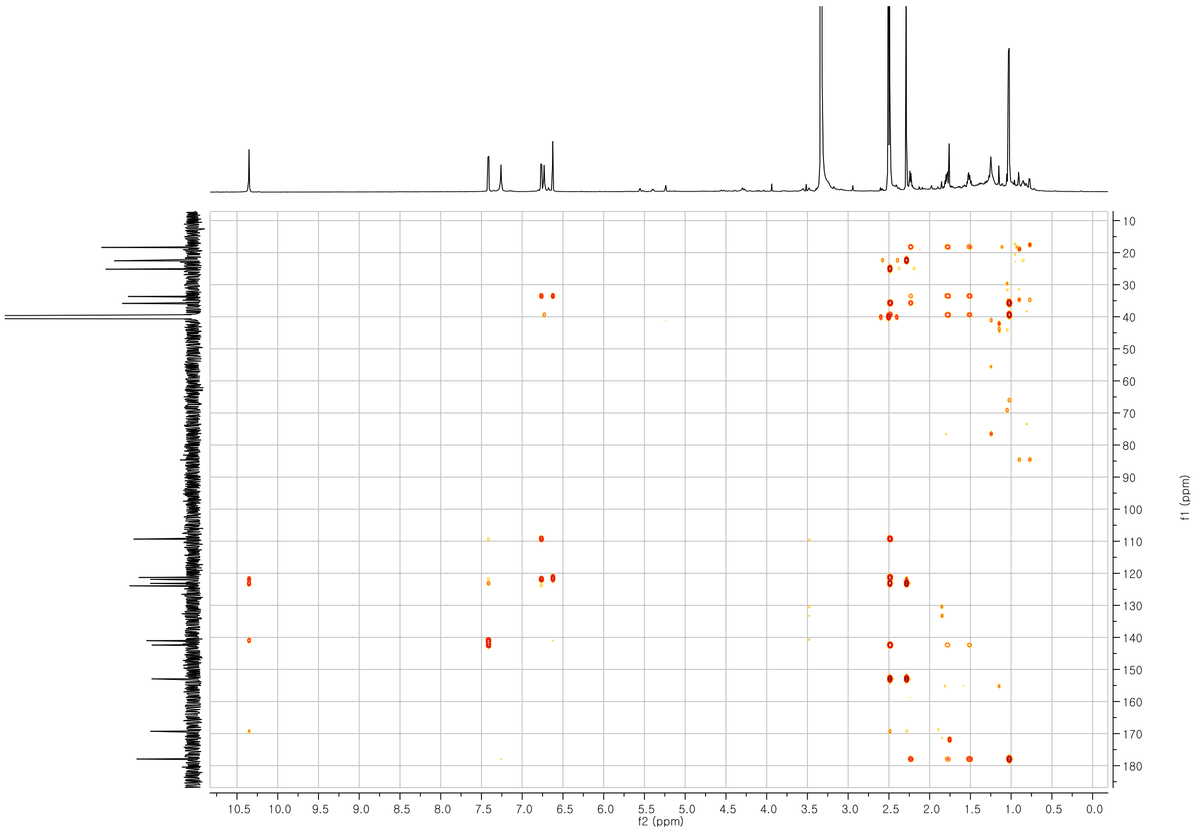

Figure S11. HMBC spectrum of RK-270E (**2**) in DMSO-d_6

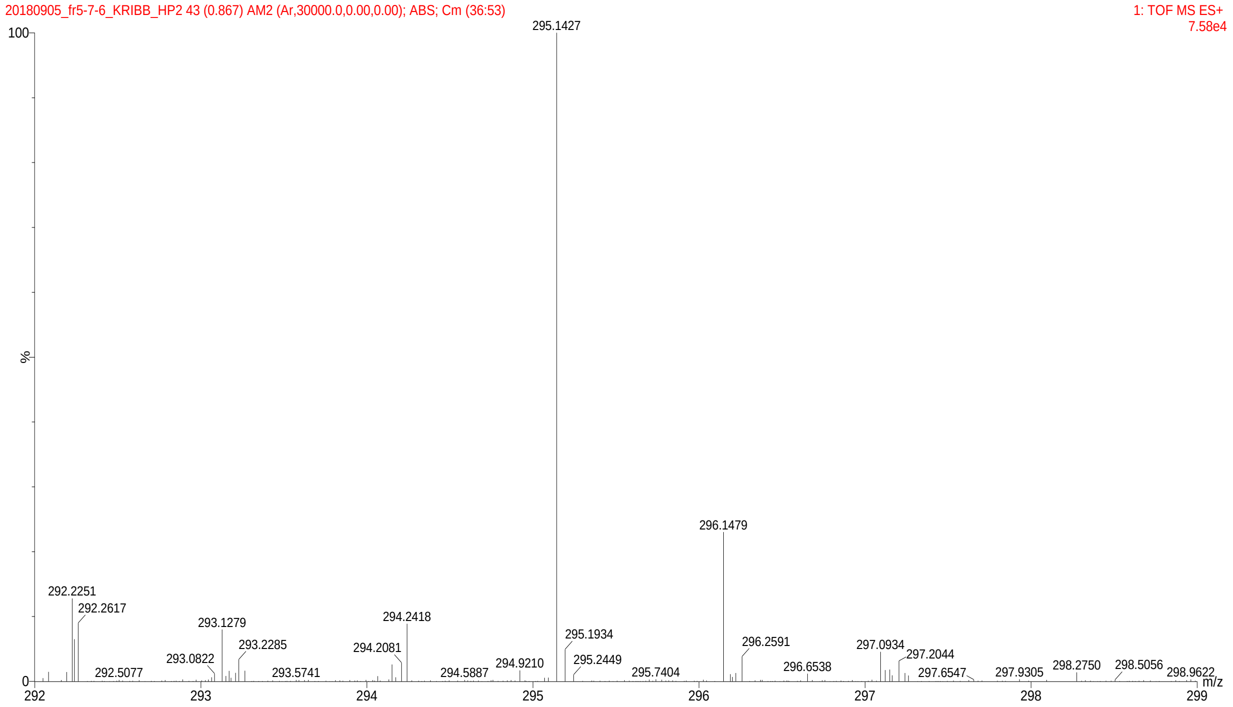

Figure S12. HRESIMS spectrum of RK-270E (**2**)

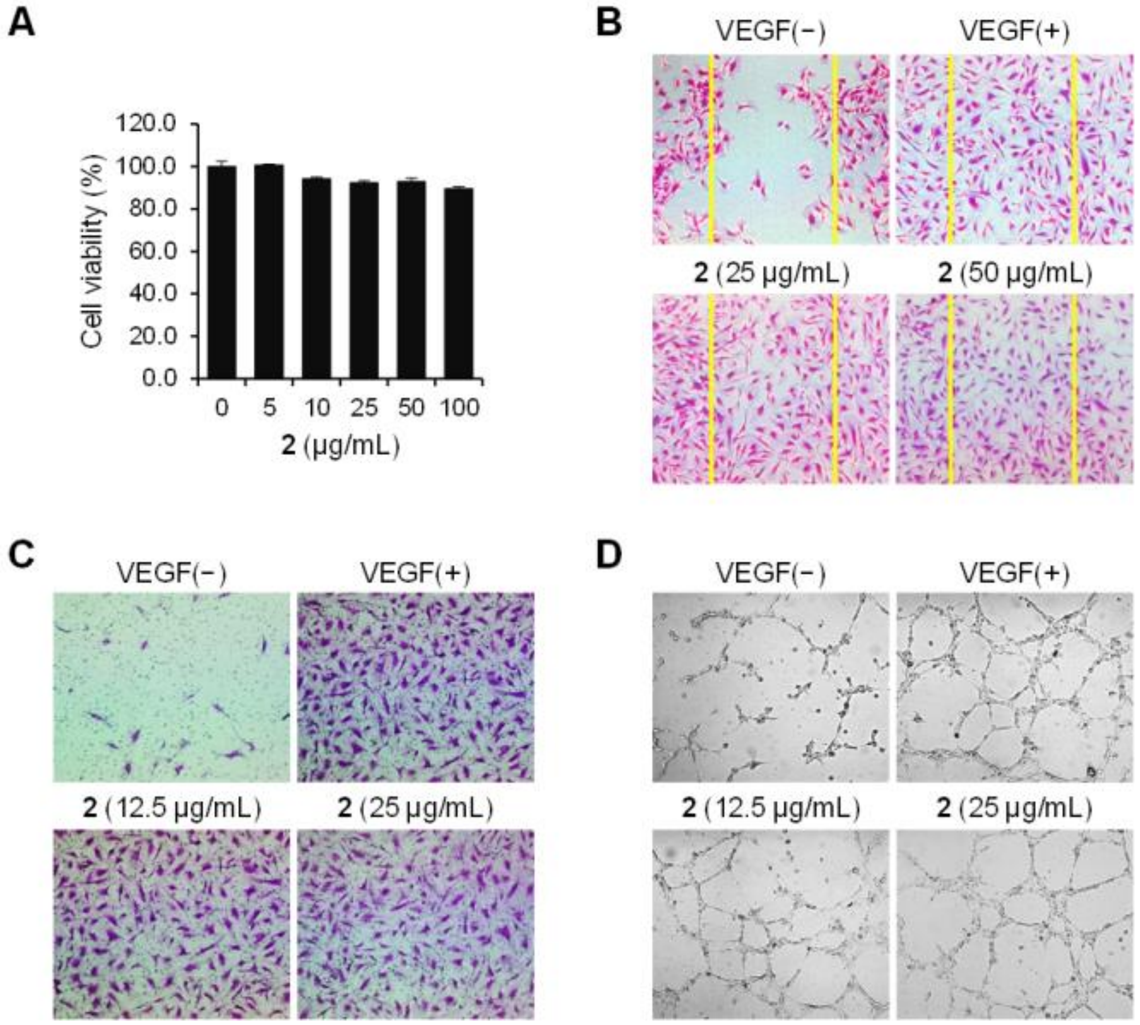

Figure S13. Effects of compound **2** on angiogenesis in HUVECs

(A) Effect of **2** on cell viability. HUVECs were treated with the indicated concentrations of **2** for 24 h. Cell viability was measured using the EZ-Cytox assay solution (mean ± SD, n = 3). (B−D) Representative images of the inhibitory effect of **2** on cell migration (B), invasion (C), and capillary tube formation (D). (B) Cells were treated with the indicated concentrations of **2** in the presence of VEGF (30 ng/mL) for 12 h and stained with crystal violet. The migrated cells were observed under a microscope. Cells not stimulated with VEGF were used as a negative control. (C) Starved cells were treated with **2** and VEGF (30 ng/mL) in the Matrigel-coated upper chamber. After 18 h incubation, the invaded cells were stained with crystal violet and imaged by a microscope. (D) Starved cells were treated with **2** and VEGF (30 ng/mL) for 3 h in the Matrigel-coated 96-well cell culture plates. Capillary-like tubular structures were captured under a microscope.

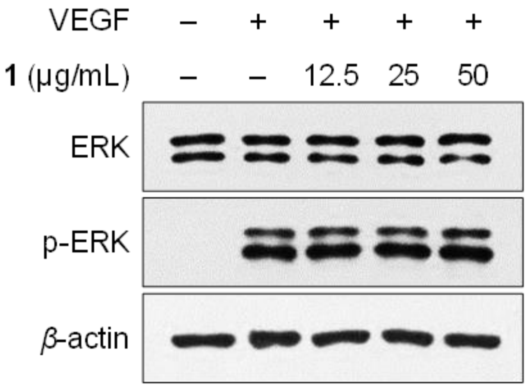

Figure S14. Effects of compound **1** on the phosphorylation of ERK in VEGF-induced HUVECs.

HUVECs were pre-treated with **1** followed by the stimulation with VEGF (30 ng/mL) for 5 min. Phosphorylation of ERK was analyzed by Western blot analysis using antibodies against ERK and p-ERK. Cells not stimulated with VEGF were used as a negative control.
